# Draft Genome Assembly of the False Spider Mite *Brevipalpus yothersi*

**DOI:** 10.1128/MRA.01563-18

**Published:** 2019-02-07

**Authors:** Denise Navia, Valdenice M. Novelli, Stephane Rombauts, Juliana Freitas-Astúa, Renata Santos de Mendonça, Maria Andreia Nunes, Marcos A. Machado, Yao-Cheng Lin, Phuong Le, Zaichao Zhang, Miodrag Grbić, Nicky Wybouw, Johannes A. J. Breeuwer, Thomas Van Leeuwen, Yves Van de Peer

**Affiliations:** a Embrapa Genetic Resources and Biotechnology, Brasília, DF, Brazil; b Sylvio Moreira Citrus Center, Agronomic Institute (IAC) Cordeirópolis, São Paulo, Brazil; c Department of Plant Biotechnology and Bioinformatics, Ghent University, Ghent, Belgium; d Center for Plant Systems Biology, VIB, Ghent, Belgium; e Embrapa Cassava and Fruits, Cruz das Almas, Bahia, Brazil and Biological Institute, São Paulo, Brazil; f Faculty of Agronomy and Veterinary (FAV), University of Brasilia (UnB), Brasília, DF, Brazil; g Department of Biology, The University of Western Ontario, London, Ontario, Canada; h Department of Plants and Crops, Ghent University, Ghent, Belgium; i Department of Evolutionary and Population Biology, Institute for Biodiversity and Ecosystem Dynamics, University of Amsterdam, Amsterdam, The Netherlands; j Department of Biochemistry, Genetics and Microbiology, University of Pretoria, Pretoria, South Africa; Indiana University, Bloomington

## Abstract

The false spider mite Brevipalpus yothersi infests a broad host plant range and has become one of the most economically important species within the genus *Brevipalpus*. This phytophagous mite inflicts damage by both feeding on plants and transmitting plant viruses. Here, we report the first draft genome sequence of the false spider mite, which is also the first plant virus mite vector to be sequenced. The ∼72 Mb genome (sequenced at 42× coverage) encodes ∼16,000 predicted protein-coding genes.

## Abstract (precis)

The false spider mite Brevipalpus yothersi infests a broad host plant range and has become one of the most economically important species within the genus *Brevipalpus*. This phytophagous mite inflicts damage by both feeding on plants and transmitting plant viruses.

## ANNOUNCEMENT

Brevipalpus yothersi Baker (Tenuipalpidae), previously misidentified as Brevipalpus phoenicis (Geijskes), was recently resurrected, redescribed, and placed in the B. phoenicis *sensu stricto* group (1). The false spider mite B. yothersi is a vector of several plant viruses, some of which cause diseases in economically important crops, such as citrus and passion fruit (2). More than 40 plant species have been reported as natural hosts of *Brevipalpus*-transmitted viruses (BTVs) (3). Despite the intense use of pesticides and acaricides (4), even low population densities of the false spider mites are sufficient to infest citrus orchards and spread diseases such as citrus leprosis (CL) in Brazil (5) and potentially in the United States and the European Union (6). Most tropical and subtropical regions in the world have resident *Brevipalpus* mites (1), and these pose a major threat to crops affected by the transmitted viruses.

Although information on the economic impact of false spider mites in agriculture is limited, it was estimated that almost 10% of the total world acaricide market value is spent on the control of *Brevipalpus* spp. (7). Brevipalpus phoenicis *sensu lato* species, which include B. yothersi (1), reproduce by thelytoky parthenogenesis, controlled by a symbiotic relationship with *Cardinium* bacteria. As a result of the reproductive manipulation, B. yothersi populations almost exclusively consist of haploid females (*n* = 2 chromosomes) (8).

For sequencing, a B. yothersi population was identified by molecular and morphological traits, as described in Navia et al. (9) and Beard et al. (1), respectively. An isofemale line was reared on sweet orange [Citrus sinensis (L.) Osbeck] fruits. For DNA extraction, 8,000 male mites, lacking *Cardinium*, were collected, flash frozen, and ground with tungsten beads in batches of 2,000 mites. Batches were homogenized, and total DNA was extracted with a DNeasy blood and tissue kit (Qiagen). The DNA samples were pooled and sequenced on a Roche 454 GS FLX+ system with one kit for unidirectional sequencing and one for the mate-pair library preparation protocol (spacing, 3 to 8 kb; reads, 700 to 1,000 bp). Additional sequencing of paired-end libraries was prepared with the Gel-Free protocol (Nextera) and performed with an Illumina MiSeq next-generation sequencer (MiSeq run, Nextera kit, 2 × 250 bp reads, 10 to 15 Gb data; see Table 1).

**TABLE 1:** Details on the Roche 454 GS FLX+ and Illumina sequencing and libraries used

Accession no.	Technology used	No. of reads	No. of nucleotides	Coverage (×)	Library type^*a*^	Molecule	Read length	Insert size
SRX4717572	Roche (454 GS FLX+)	600,711	421.8 million	5.9	SE	gDNA^*b*^	1 kb	—
SRX4676376	Roche (454 GS FLX+)	631,875	432 million	6.1	SE	gDNA	1 kb	—
SRX4676374	Illumina (MiSeq)	11.4 million	5.3 billion	74.5	PE	gDNA	250 nt^*c*^	250 nt
SRX4676377	Illumina (MiSeq)	15.7 million	4.6 billion	64.6	PE	gDNA	250 nt	250 nt
SRX4676378	Illumina (MiSeq)	2.1 million	611.3 million	8.6	MP	gDNA	250 nt	6 kb
SRX4676375	Illumina (MiSeq)	5.1 million	1.6 billion	22.5	MP	gDNA	250 nt	6 kb
SRX4676380	Illumina (MiSeq)	5.6 million	1.4 billion	19.7	MP	gDNA	250 nt	3 kb
SRX4676381	Illumina (MiSeq)	2.3 million	697.7 million	9.8	MP	gDNA	250 nt	3 kb
SRX4676379	Illumina (MiSeq)	253.5 million	50.9 billion	—	PE	RNA-seq	100 nt	200 nt

a SE, single pair; PE, paired ends; MP, mate pairs.

b gDNA, genomic DNA.

c nt, nucleotides.

Raw sequencing reads were quality trimmed, and all ends were removed with a quality Phred score below 20. The MiSeq read pairs were joined into pseudoreads and assembled with the 454 reads with an overlap-layout approach (Newbler 2.9.1). The resulting contigs were further scaffolded with the mate-pair reads (SSpace 2.0) (10), and resulting gaps were locally filled through an iterative process (GapFiller 1.10) (11). The obtained genome sequence was assembled into 3,467 contigs scaffolded into 849 larger genomic segments (*N*_50_, 632 kb; 71.18 Mb; GC, 36.8%) and was annotated with both EuGene (12) and AUGUSTUS (13). We predicted 15,929 protein-coding genes, with an average coding DNA sequence (CDS) length of 1,266 bp. The core eukaryotic protein-coding gene presence was assessed with BUSCO (14) (v3.0, 303 reference genes), with 86.5% complete orthologs present (83.2% single copy, 3.3% duplicates, 2.3% fragments, and 34 genes missing). BLASTP hits against the reference genome of the spider mite Tetranychus urticae identified 11,721 homologous genes. A search with the InterProScan tool could assign known motifs and gene ontology (GO) terms, for, respectively, 10,171 and 7,831 genes.

### Data availability.

This whole-genome shotgun project has been deposited at DDBJ/ENA/GenBank as BioProject number PRJNA490612 under the accession number QZCP00000000. The version described in this paper is the first version.
