# Erratum for Navia et al., “Draft Genome Assembly of the False Spider Mite *Brevipalpus yothersi*”

**DOI:** 10.1128/MRA.00710-19

**Published:** 2019-07-03

**Authors:** Denise Navia, Valdenice M. Novelli, Stephane Rombauts, Juliana Freitas-Astúa, Renata Santos de Mendonça, Maria Andreia Nunes, Marcos A. Machado, Yao-Cheng Lin, Phuong Le, Zaichao Zhang, Miodrag Grbić, Nicky Wybouw, Johannes A. J. Breeuwer, Thomas Van Leeuwen, Yves Van de Peer

**Affiliations:** a Embrapa Genetic Resources and Biotechnology, Brasília, DF, Brazil; b Sylvio Moreira Citrus Center, Agronomic Institute (IAC) Cordeirópolis, São Paulo, Brazil; c Department of Plant Biotechnology and Bioinformatics, Ghent University, Ghent, Belgium; d Center for Plant Systems Biology, VIB, Ghent, Belgium; e Embrapa Cassava and Fruits, Cruz das Almas, Bahia, Brazil and Biological Institute, São Paulo, Brazil; f Faculty of Agronomy and Veterinary (FAV), University of Brasilia (UnB), Brasília, DF, Brazil; g Department of Biology, The University of Western Ontario, London, Ontario, Canada; h Department of Plants and Crops, Ghent University, Ghent, Belgium; i Department of Evolutionary and Population Biology, Institute for Biodiversity and Ecosystem Dynamics, University of Amsterdam, Amsterdam, The Netherlands; j Department of Biochemistry, Genetics and Microbiology, University of Pretoria, Pretoria, South Africa

## ERRATUM

Volume 8, no. 6, e01563-18, 2019, https://doi.org/10.1128/MRA.01563-18. Page 1, line 22: “8,000 male mites, lacking *Cardinium*, …” should read “8,000 female mites, *Cardinium* infected, …”

